# MYC Rules: Leading Glutamine Metabolism toward a Distinct Cancer Cell Phenotype

**DOI:** 10.3390/cancers13174484

**Published:** 2021-09-06

**Authors:** Vincent Tambay, Valérie-Ann Raymond, Marc Bilodeau

**Affiliations:** 1 Laboratoire d’Hépatologie Cellulaire, Centre de Recherche du Centre Hospitalier de l’Université de Montréal, Montréal, QC H2X 0A9, Canada; vincent.tambay@umontreal.ca (V.T.); valerie-ann.raymond.chum@ssss.gouv.qc.ca (V.-A.R.); 2 Département de Médecine, Université de Montréal, Montréal, QC H3T 1J4, Canada

**Keywords:** oncogene, MYC, glutamine metabolism

## Abstract

**Simple Summary:**

In the last decade, metabolic reprogramming has emerged as a driving characteristic of cancer cells. The MYC oncogene, a transcription factor, has become of growing interest as a fundamental driver of differential cancer cell metabolism. Furthermore, the non-essential amino acid glutamine is deemed to be an important nutrient for cancer cells. In fact, glutamine can integrate into a wide variety of metabolic pathways, from energy metabolism to nucleotide synthesis. This review offers a comprehensive and specific overview of recent discoveries in the regulation of MYC oncogene activation on glutamine metabolism in cancer cells.

**Abstract:**

Metabolic reprogramming and deregulated cellular energetics are hallmarks of cancer. The aberrant metabolism of cancer cells is thought to be the product of differential oncogene activation and tumor suppressor gene inactivation. MYC is one of the most important oncogenic drivers, its activation being reported in a variety of cancer types and sub-types, among which are the most prevalent and aggressive of all malignancies. This review aims to offer a comprehensive overview and highlight the importance of the c-Myc transcription factor on the regulation of metabolic pathways, in particular that of glutamine and glutaminolysis. Glutamine can be extensively metabolized into a variety of substrates and be integrated in a complex metabolic network inside the cell, from energy metabolism to nucleotide and non-essential amino acid synthesis. Together, understanding metabolic reprogramming and its underlying genetic makeup, such as MYC activation, allows for a better understanding of the cancer cell phenotype and thus of the potential vulnerabilities of cancers from a metabolic standpoint.

## 1. What Is the MYC Oncogene?

Through decades of research, the revolution in molecular biology has been intimately linked with advancements in oncogenetics. This global interest has led to the discovery of genes that promote the development of cancer and their physiological counterparts, respectively called oncogenes and proto-oncogenes. The v-Myc oncogene was first identified in 1964 as the transforming element of the MC29 retrovirus that was isolated from a chicken having developed spontaneous myelocytomatosis [1,2]. Myelocytomatosis is characterized by the formation of foci of eosinophilic promyelocytes and metamyelocytes in bone marrow [2]. Further studies in the 1970s characterized that the MC29 retrovirus promoted a spectrum of malignancies, including endotheliomas, sarcomas, and importantly, carcinomas such as those of the liver and kidneys [1,2]. The discovery of the cellular MYC oncogene, also termed c-Myc, further reinforced the notion that oncogenic transformation could be caused by the activation of cellular genes [2].

**Figure 1:**
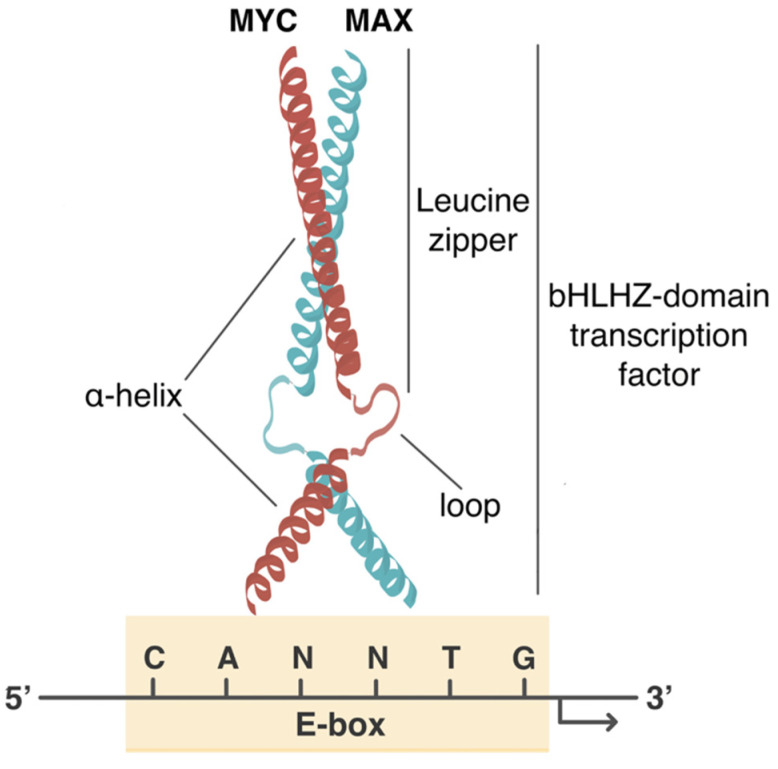
Molecular interactions of MYC: dimerization with MAX and DNA binding. MYC is a basic helix-loop-helix leucine-zipper (bHLHZ) transcription factor. The helix-loop-helix domain allows DNA binding, whereas leucine zipper allows the MYC–MAX interaction. MYC transcription factor requires dimerization with MAX to recognize and bind to specific DNA CANNTG sequences (where N can be any nucleotide), termed E-box.

The c-Myc protein is a basic helix-loop-helix leucine zipper (bHLHZ) family transcription factor that executes its function by dimerizing with the small bHLHZ protein Myc-Associated factor X (MAX) (Figure 1). This interaction is required for c-Myc to recognize specific DNA elements and recruit transcriptional co-regulatory molecules to regulate the expression of a broad but selective ensemble of genes [3,4,5]. The c-Myc-MAX heterodimer directly activates the transcription of its target genes by binding to specific DNA sequences [5]. Among these is the E-box, CANNTG (where N can be any nucleotide), with CACGTG being the palindromic canonical consensus DNA binding site of c-Myc [3,6]. c-Myc can also bind several non-canonical sequences to regulate gene expression [6]. c-Myc activity is also controlled by a complex network of co-regulatory proteins, named the proximal MYC network (PMN) [4]. The PMN includes several proteins that bear bHLHZ domains, whether they be stimulatory, such as MAX, or inhibitory of c-Myc activity [4]. Interestingly, studies have reported that repressor members of the PMN act as tumor suppressors and consequently bear deletions in certain tumor types [4,7,8].

## 2. MYC-Related Genetic Alterations in Cancer

In normal cells, c-Myc acts as a transcription factor that responds strictly to mitogenic and cellular developmental signals by integrating differential gene expression, supporting cell growth and proliferation in response to physiological stimuli [4]. However, during tumorigenesis, genetic alterations give rise to increased and deregulated c-Myc protein expression, rendering the latter insensitive to normal cellular signals and regulatory constraints [4]. An example of MYC gene alterations are point mutations that result in increased c-Myc protein stability and activity, as identified in the c-Myc-activating translocations in Burkitt’s lymphoma [4,9]. Other genetic alterations leading to c-Myc upregulation include MYC enhancer amplification, which are rare events, as well as activating mutations in signal transduction pathways, such as Wnt/β-catenin [4,9]. Indeed, Rennoll et al. have shown that c-Myc expression is influenced by Wnt/β-catenin signaling in colorectal carcinoma [10]. That is, the β-catenin/TCF-LEF transcription factor complexes bind to the Wnt response elements located in the MYC gene [10]. In human gastric carcinoma cells, the circular RNA circHECTD1 has been shown to enhance c-Myc through the suppression of miR-1256, which inhibits the ubiquitin-specific peptidase 5, an upstream activator of the β-catenin signaling pathway [9]. Moreover, KRAS, the most frequently mutated oncogene during carcinogenesis, is a potent regulator of c-Myc expression and stability [11,12,13]. KRAS mediates c-Myc activity through the following two main pathways: the ERK pathway, downstream of KRAS, stabilizes c-Myc proteins by phosphorylation at Ser62 and the KRAS effector pathway PI3K-Akt inhibits GSK-3β, a known repressor of c-Myc [11,12,13]. In fact, the expression and activity of c-Myc have been reported to be crucial for the development and growth of mutant KRAS-driven pancreatic ductal adenocarcinoma (PDAC) [12,14]. Altogether, the dysregulation and overexpression of c-Myc enables its binding to lower affinity sites in gene promoters, resulting in the ectopic regulation of thousands of genes [4]. Thus, although under physiological conditions, the c-Myc-MAX heterodimer targets a specific set of genes, the selectivity of transcriptional targets is often compromised in the context of tumorigenesis [4]. While the genetic alterations that bring about c-Myc overexpression in cancer cells are diverse, the most frequent genetic alterations related to MYC are gains in gene copies [4].

Alterations in MYC genetics have been described in a variety of cancer types [4]. For example, MYC amplifications are reported in as many as 48% of breast cancers and as many as 78% of osteosarcomas [4]. In fact, copy gains account for the most common genetic alterations resulting in c-Myc overexpression [4]. According to The Cancer Genome Atlas (TCGA), MYC amplification occurs in 21% of all tumor samples, whereas that of the MYCN and MYCL paralogs are much less frequent [4]. MYCN overexpression was first identified in neuroblastomas and MYCL in lung tumors, hence each of their names [15,16]. Together, MYCN, MYCL, and MYC, as paralogs, contain regions of structural homology but remain distinct oncogenes located on chromosomes 2p24.3, 1p34.2, and 8q24.21, respectively [15,17]. The focal amplifications of MYC occur most frequently in ovarian serous cystadenocarcinoma (64.8%), followed by esophageal carcinoma (45.3%), lung squamous cell carcinoma (37.2%), uterine carcinosarcoma, and bladder urothelial carcinoma [4]. Tumor types, such as stomach adenocarcinoma, lung adenocarcinoma, breast invasive carcinoma, and hepatocellular carcinoma, also exhibit frequent MYC amplification, but not that of MYCN and MYCL gene paralogs [4]. Interestingly, among the 33 cancer types studied in TCGA, only thymoma, thyroid carcinoma, chromophobe renal cell carcinoma, acute myeloid leukemia, papillary renal cell carcinoma, and pheochromocytoma/paraganglioma have infrequent amplifications of either of the three MYC paralogs [4]. Although generally being a less common occurrence, mutations in c-Myc-antagonizing PMN members, such as MGA and MXI1, are submitted to null or hypomorphic mutations, especially in hepatocellular carcinoma, lung adenocarcinoma, and uterine carcinosarcoma [4]. 

## 3. MYC Oncogene Activation and Glutamine Addiction

**Figure 2:**
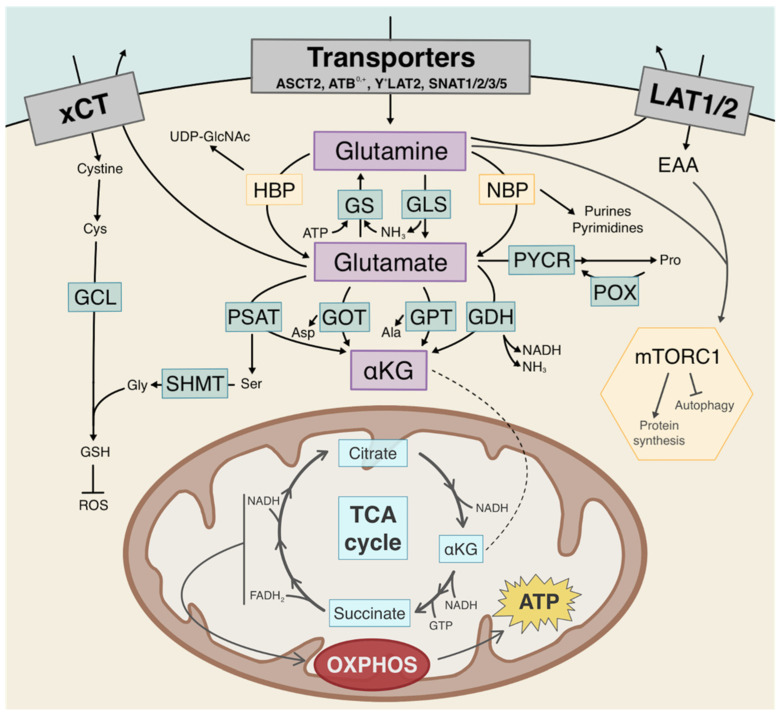
Intracellular and metabolic pathways of glutamine. Glutamine can integrate into a wide variety of metabolic and intracellular pathways, such as hexosamine biosynthesis (HBP), nucleotide synthesis (NBP), and mitochondrial metabolism through conversion to glutamate by glutaminase (GLS), and further to α-ketoglutarate (αKG) by glutamate dehydrogenase (GDH) or transamination (PSAT/GOT/GPT). Glutamine can be synthetized endogenously by glutamine synthetase (GS). Through glutamate, glutamine integrates the synthesis of many amino acids such as alanine, aspartate, serine, glycine (SHMT), and proline (P5CS). Glutamine can be exchanged for essential amino acids (EAA) through the LAT transporters, which, in turn, stimulate the mechanistic Target of Rapamycin Complex 1 (mTORC1) signaling pathway. Additionally, glutamate can be exchanged for cystine by xCT, upstream of glutathione (GSH) synthesis through glutamate-cysteine ligase (GCL).

Glutamine is the most abundant amino acid in blood circulation and can integrate a wide variety of metabolic pathways inside the cell [18] (Figure 2). In 2007, Yuneva et al. were among the first to characterize that MYC-driven proliferative cells exhibited a glutamine-addicted phenotype [19]. In fact, lung IMR-90 fibroblasts had different sensitivities to glutamine withdrawal in vitro when cells were MYC-activated by 4-hydroxytamoxifen (4-OHT) compared to untreated, MYC-inactive fibroblasts [19]. Glutamine deprivation accelerated fibroblast cell death, but only for those with an active overexpression of MYC [19]. In MCF10A mammary epithelial cells, glutamine deprivation followed by the activation of c-Myc led to increased apoptosis [20]. Interestingly, glucose deprivation of IMR-90 cells, as well as Burkitt’s lymphoma Ramos and Raji cells, did not correlate with increased apoptosis [19,21]. Likewise, c-Myc-dependent sensitivity to glutamine deprivation, termed glutamine addiction, has also been described in glioma SF188 cells and can be repressed by molecularly targeting MYC expression [22]. Since then, an addiction to extracellular glutamine for cell survival and growth under MYC oncogene activation has been demonstrated in a variety of cells from different tumor types, namely by Dejure et al. in HCT116 colorectal carcinoma cells [23], Anso et al. in osteogenic sarcoma cells [24], Gao et al. in human B lymphoma P493-6 and human prostate cancer PC3 cells [25], Jeong et al. in Ramos and Raji cells [21], Thorén et al. in human small cell lung cancer U-1906 cells [26], Shroff et al. in isolated c-Myc-driven murine renal-cell carcinoma cells [27], as well as by Haikala et al. in human fibrocystic mammary epithelial cells [20]. Moreover, studies focusing on KRAS, a key gene involved in c-Myc protein stability and activity, suggest that the phenomenon of glutamine addiction extends from the context of the MYC. In cancer cells, KRAS mutations have been shown to give rise to a dependency on exogenous glutamine for growth and proliferation, similar to that observed in MYC amplification [28,29,30].

Interestingly, recent studies have found that glutamine itself acts as a relevant regulator of c-Myc protein expression. In HCT116 cells, a concomitant decrease in c-Myc expression was observed when the cells were deprived of glutamine, which was restored by replenishing the cellular medium with glutamine [23]. This was similarly observed in various cell lines such as U266 and INA-6 multiple myeloma cell lines [31] as well as neuroblastoma SK-N-AS and SH-SY5Y cells [32]. Dejure et al. sought to further understand the mechanism through which glutamine regulated c-Myc expression. Pulse-labeling cells with ^35^S-Methionine showed that glutamine restriction shut down the translation of MYC transcripts in a mTORC1-independent manner [23]. Furthermore, glutamine starvation led to the inclusion of the 3′ untranslated transcribed region (UTR) of MYC transcripts, thus downregulating c-Myc proteins [23]. Only the addition of extracellular adenosine or glutamine could restore endogenous adenosine, which, in turn, re-established c-Myc expression [23]. Therefore, the differential expression of c-Myc under glutamine deprivation was explained by the ability of its 3′ UTR to sense adenosine, specifically downstream of glutamine [23]. Effenburger et al. confirmed that glutamine regulates c-Myc post-transcriptionally, as MYC mRNA levels remained unaltered in the absence of extracellular glutamine, whereas the proteasomal degradation of c-Myc proteins was inhibited [31]. Furthermore, Yue et al. reported that the exchange of glutamine for essential amino acids (EAA) through the LAT1 exchanger was crucial for MYC mRNA translation, since the depletion of LAT1 led to an intracellular amino acid shortage, thus activating the integrated stress response pathway via general control non-repressed-2 (GCN2) [5]. In fact, GCN2 activation phosphorylates the translation initiation factor eIF2A to adapt the translation activity during amino acid deprivation, meaning that the loss of glutamine as an exchanger for EAA halted c-Myc translation [5].

Overall, the current state of findings proposes a cellular phenotype in which c-Myc-overexpressing proliferative cells are addicted to exogenous glutamine for survival and growth. Furthermore, glutamine acts as an indirect regulator of c-Myc expression through diverse post-transcriptional pathways. 

## 4. c-Myc’s Regulation of Intracellular Glutamine Synthesis

Glutamine, being a non-essential amino acid (NEAA), can be produced endogenously through an ATP-dependent process [33,34,35]. Glutamine synthesis is accomplished by the glutamate-ammonia ligase, also termed glutamine synthetase (GS) [33,34,35]. This endogenous synthesis of glutamine from glutamate allows cells to better fulfill their biosynthetic demands by incorporating glutamine-derived amide groups in a wide variety of intracellular substrates, such as hexosamines, NEAAs, and nucleotides [34,36]. Although a conditional dependence on the exogenous supply of glutamine has been reported in MYC-overexpressing cells, the endogenous production of glutamine could remain in the cell’s best interest as it supports the flux of glutamine through many metabolic pathways [36]. In fact, GS is a downstream target of a major MYC regulator, the Wnt/β-catenin pathway [37]. Of particular interest, c-Myc has been shown to interact with the expression of GS (GLUL gene) by two independent groups [3,38].

According to Bott et al., the mRNA and protein levels as well as the enzymatic activity of GS increased in a c-Myc-dependent manner in mouse fetal liver FL5.12 Akt/Myc and MCF10A cells [3]. GLUL transcription was induced by c-Myc through TET3 and TDG, two epigenetic modifiers, by binding to the TDG promoter at two distinct E-boxes [3]. TET3, a methyl-cytosine dioxygenase, and TDG, thymine DNA glycosylase, work together to actively demethylate DNA, where TET3 oxidizes 5-methylcytosine and TDG removes the latter by base excision repair [3,39]. Thus, c-Myc indirectly increased GLUL transcription through the TDG and TET3-dependent demethylation of the GLUL promoter region [3]. Accordingly, this c-Myc-dependent increase in cellular glutamine synthesis was used to support nucleotide synthesis and the uptake of EAA [3]. 

On the other hand, Yuneva et al. reported observations opposite to those previously discussed. In MYC-induced FVB/N mouse liver tumors, GLUL mRNA and protein were low relative to the adjacent normal tissue [38]. Glutamine levels remained undetectable in MYC-driven tumors, whereas they were increased in other liver tumor types, such as those driven by the MET oncogene, which was supported by the increased syntheses of glutamine-derived glutamate, aspartate, citrate, and lactate, suggesting that MYC drives extensive glutamine metabolism, through the tricarboxylic acid (TCA) cycle and lactic acid production [38]. Contrary to liver tumors, MYC-driven lung adenocarcinomas exhibited elevated levels of glutamine as well as GS protein expression, proposing that both the tissue of origin and the genetic makeup determine the metabolic profile of tumors [38].

Together, these studies suggest the differential expression and activity of GS in the context of MYC oncogene activation. However, further investigations are required to provide a better understanding of the negative or positive correlations between MYC and glutamine synthesis.

## 5. c-Myc’s Regulation of Glutamine Uptake, Transport, and Consumption Rate

As discussed previously, c-Myc-overexpressing cells exhibit a specific and important dependency to exogenous glutamine. As such, glutamine consumption has been reported to depend on MYC in SF188 cells [22]. The same has been observed in breast cancer cells as well as in HO15.19 fibroblasts, in which c-Myc indirectly upregulates glutamine uptake by targeting the expression of glutamine transporters [40]. In HCC-3 liver tumor cells that overexpress c-Myc, Liu et al. assessed the uptake of all amino acids, essential and non-essential, and found that the most drastic increase under c-Myc was that of glutamine [41]. Hence, many studies have endeavored to identify the mechanisms that bridge c-Myc overexpression and activity to cellular glutamine uptake. Glutamine, although being nonessential, can be transported into the cell through many transporters having various physiological mechanisms, such as ASCT2, ATB^0,+^, Y^+^LAT2, SNAT1, SNAT2, SNAT3, and SNAT5 [42]. Interestingly, many of these transporters have been linked with human diseases, such as ASCT2, a high affinity glutamine transporter detected in several malignancies [42,43].

Firstly, Wise et al. showed that the ASCT2 and SNAT5 mRNA levels were dependent on MYC expression in SF188 cells [22]. Incidentally, c-Myc, through its role as a transcription factor, increased both ASCT2 and SNAT5 transcription by binding at canonical E-boxes in each of their promoters [22]. RNA sequencing analyses in U266 cells have confirmed that the cells subject to MYC activation upregulate SNAT5 mRNA [31]. The influence of c-Myc on ASCT2 transcript levels has been further confirmed by Chen et al. in breast cancer LTEDaro cells [44], Thorén et al. in U-1906 cells [26], Wu et al. in colorectal carcinoma DLD-1 cells [45], and Liu et al. in HCC-3 cells [41]. Likewise, the c-Myc-dependent increase in ASCT2 mRNA expression was accompanied by a concomitant increase in protein expression in human colorectal carcinoma SW620 cells [46]. Furthermore, the pharmacological inhibition of c-Myc with the 10058-F4 small molecule in FaDu hypopharyngeal carcinoma cells confirmed that ASCT2 expression is dependent on the activity of c-Myc [47]. These findings have also been corroborated by in vivo studies, where the induction of renal cell carcinoma in mice through MYC oncogene activation resulted in increased ASCT2 and SNAT1 but decreased SNAT2 mRNA levels [27]. The differential expression of the SNAT1 transporter has also been proven to be downstream of c-Myc, namely in DLD-1 cells through binding at the transcription start site [48] and in murine T lymphocytes at both mRNA and protein levels [49]. Furthermore, Liu et al. have shown that the glutamine uptake in HCC-3 cells is accomplished exclusively by ASCT2 and Y^+^LAT2 [41]. Specifically, the Y^+^LAT2 mRNA and protein upregulation was dependent on c-Myc-driven transcription by binding at the Y^+^LAT2 promoter region [41].

Besides its integration in a wide variety of metabolic and cell signaling pathways, glutamine can be used as an exchanger for the uptake of other amino acids, notably EAAs. Two transporters with a high affinity for glutamine, tyrosine, and all EAAs but lysine, are LAT1 and LAT2 [42,50]. As obligatory exchangers, the latter are thought to potentially support the influx of EAAs coupled with the efflux of NEAAs such as glutamine [42,50]. In MycER-expressing P493-6 cells, the uptake of EAA, such as leucine and phenylalanine, has been shown to be regulated by c-Myc [5]. In fact, Yue et al. demonstrated the transcriptional ability of c-Myc to bind the LAT1 promoter, as shown in P493-6, HEK293T, and neuroblastoma BE-2C cell lines [5]. Similar observations were reported in rat embryonic fibroblasts, mouse tail fibroblasts, and cultured primary mouse hepatocytes, and further confirmed in vivo in MYC hypomorphic and wild-type mice [40]. Likewise, LAT1 mRNA and protein levels were upregulated by the genic activation of MYC in isolated T lymphocytes [49]. On the other hand, one study has shown that LAT2 mRNA is enriched when MYC is expressed in U266 cells [31].

Altogether, c-Myc is responsible for regulating the expression of many genes involved in glutamine and amino acid transport. These findings integrate the metabolic reprogramming of glutamine metabolism in both neoplastic and non-neoplastic contexts, where MYC-driven cells require an increased glutamine and EAA uptake, which is, in turn, supported by c-Myc activity.

## 6. c-Myc’s Regulation of Glutaminase-Mediated Glutaminolysis

Glutaminolysis is the process by which glutamine is converted to glutamate to fuel the TCA cycle [18]. Consequently, cells can use glutamine as a substrate for energy metabolism and mitochondrial respiration [51]. Glutaminolysis is accomplished by the glutaminase enzyme, a hydrolase that breaks down glutamine into glutamate, while liberating free ammonia as a byproduct [18,34,51]. Thus, similar to lactate, a byproduct of rapid glucose metabolism, ammonia is the product of glutamine breakdown [51]. Glutaminase (GLS) exists as two distinct isoforms, the kidney-type GLS1, expressed in most extrahepatic tissues and expressed in multiple malignancies, as well as the liver-type GLS2, specific to hepatocytes [34,52].

As a classic measure for the rate of glutamine breakdown, various studies have assessed ammonia production in the context of MYC oncogene activation. For example, in SF188 cells, ammonia production was shown to be dependent on MYC expression [22]. Similarly, in FaDu cells, the pharmacological inhibition of c-Myc suppressed ammonia production by 60%, suggesting a drastic decrease in glutaminolysis [47]. Similar findings have been reported in P493-6 cells [53]. Furthermore, metabolite profiling has shown that glutamine is among the most depleted metabolites in MYC-driven liver tumors, whereas glutamate, the product of the first step of glutaminolysis, is abundant in tumor tissue compared to normal liver, which could be explained by the MYC-dependent upregulation of GLS [54]. The induction of c-Myc in mouse embryonic fibroblasts has been shown to increase GLS1 mRNA [22]. Likewise, GLS1 protein expression was increased as a response to MYC activation in P493-6 and PC3 cell lines [25]. Similar observations have been reported in SW620 cells [46], U-1906 cells [26], MYC-induced murine liver tumors [38,55], lung adenocarcinoma [38], murine renal cell carcinoma [27], human hepatocellular carcinoma [56], as well as in MYC-activated U266-derived cells [31]. Furthermore, both GLS1 and GLS2 transcript levels have been shown to be dependent on c-Myc expression in colorectal carcinoma DLD-1 cells [45]. Interestingly, the same was found to be true for GLS1 in liver tumors, but not for GLS2, which is, as previously stated, the hepatocyte specific GLS [38,55]. GLS2 proteins were also downregulated in MYC-induced renal cell carcinoma [27]. Conversely, in T lymphocytes, GLS2 mRNA and protein expression was supported by c-Myc [49]. Together, the current state of the literature suggests that c-Myc is a relevant upregulator of GLS1 expression. However, further studies are required to better understand the association between c-Myc and GLS2 expression, as current findings are contradictory.

Studies have aimed to explain the mechanisms through which c-Myc regulates glutaminolysis by mediating GLS upregulation. First of all, Gao et al. did not observe any evidence of transcriptional regulation of GLS1 by c-Myc, despite having a canonical E-box sequence in its first intron [25]. Rather, in PC3, P493-6, human lymphoid CB33, and breast cancer MCF-7 cells, c-Myc regulated GLS1 expression in a post-transcriptional manner, implicating the microRNAs miR-23a and miR-23b [25]. In fact, c-Myc suppressed miR-23a/b, which were found to repress GLS1 translation by binding the 3′UTR of GLS1 transcripts [25]. For miR-23b, c-Myc was reported to directly bind the chromosome 9 open reading frame 3 transcriptional unit, c9orf3, that encompasses mi-R23b [25]. On the other hand, Haikala et al. proposed that the influence of c-Myc on GLS1 expression occurs through transcriptional regulation by binding to the GLS1 promoter at the transcription start site near the 5′ UTR [20]. Thus, in addition to being indirectly regulated through miR-23a/b, GLS1 expression can also be a direct transcriptional target of c-Myc [20].

Lastly, GLS1 expression and activity were found to be crucial for the rapid proliferation of MYC-amplified cells, such as P493-6 and PC3 cell lines, in which interfering with GLS1 expression attenuated cell proliferation and had similar effects to glutamine deprivation [25]. In MYC-induced murine renal carcinoma cells, the pharmacological inhibition of GLS1 by BPTES slowed tumor progression [27]. Similarly, in MYC-induced murine hepatocellular carcinoma, GLS1^+/−^ heterozygotes had smaller tumor loads than their wild-type GLS1^+/+^ counterparts [55]. Overall, these findings are strong evidence that GLS1 is essential for the optimal tumorigenesis, tumor progression, and cancer cell proliferation driven by the MYC oncogene.

## 7. c-Myc’s Regulation of Glutamate Metabolism: Deamination and Transamination

Once glutamine is converted into glutamate, the latter is further metabolized into α-ketoglutarate (αKG). The deamination of glutamate, that is the subsequent step after glutaminolysis, is executed through two main pathways. The first is through glutamate dehydrogenase (GLUD), which removes the amine group from glutamate [57,58]. As a result, only the carbon skeleton of glutamine remains, αKG, while free ammonia and NADH are released as byproducts [57,58]. The second pathway is transamination, which refers to the transfer of the amine group from glutamate to synthetize other NEAAs, instead of releasing this amine as free ammonia [18,59]. The transamination of glutamate yielding αKG is performed by three distinct transaminases or aminotransferases [18,59]. The first, glutamic-oxaloacetic aminotransferase (GOT), aminates oxaloacetate to form aspartate [18]. The second, glutamic-pyruvic aminotransferase (GPT), forms alanine from pyruvate [18]. Both the GOT and GPT transamination reactions occur in the cytosol and the mitochondrion, with organelle-specific isoforms one and two, respectively [18]. The third is the phospho-serine aminotransferase (PSAT1), responsible for aminating 3-phosphohydroxypyruvate into O-phospho-L-serine, the direct precursor to L-serine [18,60]. These three pathways lead to αKG, a TCA cycle intermediate, which enables glutamine to integrate mitochondrial metabolism.

The influence of c-Myc on the expression of glutamate dehydrogenase and glutamate-dependent transaminases has been reported by various independent groups aiming to characterize c-Myc-driven metabolic reprogramming in different tumor types. First off, the GLUD1 protein levels have been characterized as being elevated in MYC-induced murine renal cell carcinoma [27]. The GLUD1 transcript levels have also been found to be dependent on c-Myc during T lymphocyte activation [49]. On the other hand, Korangath et al. reported that the GLUD1 mRNA levels were impacted by c-Myc knockdown to a much lesser extent than the transaminases in triple negative breast cancer SUM159 cells [61]. For alanine transaminases, only mitochondrial GPT2 has been shown to be strongly suppressed at the transcript level as a result of c-Myc loss in SUM159 cells [61]. The same was observed at the protein level in osteogenic sarcoma during c-Myc suppression, which in turn leads to a return to osteocyte differentiation [24]. GOT1 was also upregulated by MYC activation at the protein level in renal cell carcinoma in mice, as described by Shroff et al. [27]. The same has been observed for GOT2 at the mRNA level in Burkitt’s lymphoma Daudi cells [5] and SUM159 cells [61] as well as GOT2 protein in osteogenic sarcoma [24]. Incidentally, the KRAS oncogene has been proven to reprogram glutamate metabolism preferentially through GOT1 rather than GLUD1 in PDAC [28,62]. Finally, concerning PSAT1, both the mRNA and protein expressions were dependent on c-Myc in Burkitt’s lymphoma Raji and NAMALWA cells [63], P493-6 cells, and human hepatocellular carcinoma Hep3B cells [64]. 

The mechanisms through which c-Myc regulates the expression of GLUD1, GPT2, GOT1, GOT2, and PSAT1 genes remain unclear. Notwithstanding, the positive correlations observed between the mRNA expression of these five genes and c-Myc suggest that the latter acts at least transcriptionally. This has been demonstrated by Sun et al., who showed the direct binding of c-Myc to E-box sequences near the transcription start site of the PSAT1 promoter in Hep3B and P493-6 cells [64]. Another explanation, proposed by Białopiotrowicz et al., supports the ability of c-Myc to regulate the activating transcription factor 4 (ATF4) that, in turn, increases PSAT1 expression by binding to its promoter region [63].

Furthermore, the transamination pathway has also been shown to be of particular interest in understanding the fate of glutamine during c-Myc-driven metabolic reprogramming. For example, in P493-6 cells, glutamine was highly incorporated into glutamate, alanine, and aspartate in MYC-overexpressing cells [65]. Interestingly, glutamate transamination downstream of the first step of glutaminolysis has been shown to be crucial for c-Myc-amplified cell survival and proliferation in various malignancies. Wise et al. have shown that SF188 cells are sensitive to the inhibition of the transamination pathway by aminooxyacetate (AOA), which induces apoptosis [22]. The same observation was made in SUM149 and SUM159 triple negative breast cancer cells; the sensitivity to AOA was further found to be specific to MYC-active cells [61]. Additionally, the sensitivity to AOA treatment has been described as a hallmark of mutant KRAS in cancer cells, which cooperates with MYC in cancer metabolic reprogramming [62,66]. In P493-6 cells, the loss of the expression of either GOT1, GOT2, or GPT1 in the context of MYC amplification has been shown to suppress cell proliferation [65]. Furthermore, AOA treatment decreased cell viability and mitochondrial respiration in SF188 and osteogenic sarcoma cells but were both restored by cell-permeable dimethyl-αKG, αKG being an important product of transamination [22,24]. This suggests that Myc-driven tumor cells preferentially use transamination to support αKG production from glutamine for mitochondrial metabolism. On the other hand, Jeong et al. observed that both Ramos and Raji cells were dependent on GLUD activity for proper glutamine consumption and metabolism [21]. These findings together support the importance of GLUD, in addition to transamination, in MYC-dependent glutamine metabolism in cancer cells.

## 8. c-Myc’s Regulation of Glutamine-Dependent Mitochondrial Activity

The deamidation of glutamine and the subsequent deamination of glutamate to αKG support energy metabolism in the cell. Indeed, energy-demanding cells require TCA cycle intermediates, such as αKG, to be replenished, a process termed anaplerosis [67,68]. αKG, as previously discussed, is the product of glutaminolysis [68,69]. Hence, glutamine, through αKG, supports TCA cycling that, in turn, produces the NADH and FADH_2_ coenzymes used to produce ATP through oxidative phosphorylation [51,70].

The glutamine-dependent TCA cycle anaplerosis with subsequent mitochondrial respiration has been reported to be a fundamental characteristic of MYC-driven metabolism. For example, Feist et al. have shown that, under glutamine deprivation, P493-6 cells have drastically reduced oxygen consumption rates [65]. Oxygen consumption rates were also substantially increased in c-Myc-overexpressing osteogenic sarcoma cells compared to MYC-inactive osteocytes [24]. Such findings suggest that mitochondrial activity and oxidative phosphorylation is dependent on glutamine in the context of c-Myc-reprogrammed metabolism. In fact, glutamine carbon labeling by three independent studies has shown that c-Myc activation fuels the TCA cycle by driving glutamine toward αKG, as seen by the increased levels of glutamine-derived succinate, fumarate, and malate [24,53,71]. Moreover, osteogenic sarcoma cells shift from glutamine-dependent TCA cycle anaplerosis to reductive carboxylation during the loss of c-Myc expression [24]. These studies together confirm that c-Myc promotes glutamine-derived anaplerosis of the TCA cycle. Importantly, under c-Myc activation, the replenishing of TCA cycle substrates has been shown to be specific to glutamine metabolism, that of glucose not being involved. Precisely, in SF188 cells, c-Myc activation led to the Warburg phenotype, in which glucose is diverted away from mitochondrial metabolism and preferentially metabolized into lactate [22]. Interestingly, Kleszcz et al. found that the inhibition of c-Myc using 10058-F4 in FaDu cells reversed the glutamine-dependent phenomenon of TCA cycle anaplerosis and instead increased glucose flux to the mitochondria [47].

**Figure 3:**
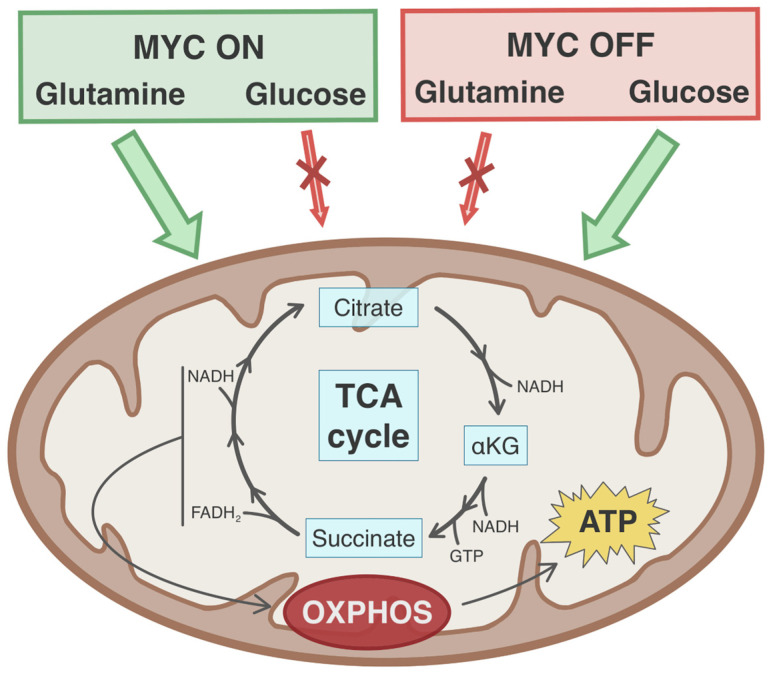
TCA cycle anaplerosis under the MYC oncogene. On the left (green box), activation of the MYC oncogene drives preferential metabolism of glutamine toward TCA cycle anaplerosis and subsequent oxidative phosphorylation. On the right (red box), loss of MYC oncogene expression increases dependency on glucose for mitochondrial metabolism.

Additionally, to understand how c-Myc rewires mitochondrial activity to be dependent on glutaminolysis, Fogal et al. showed that the p32 mRNA and protein levels were induced by c-Myc in MRC5 human pulmonary fibroblasts [6]. The p32 protein supports mitochondrial oxidative phosphorylation by playing a role in the synthesis of electron transport chain complex subunits and has been suggested to play a role in tumorigenesis [6,72]. Incidentally, p32 was found to be a direct transcriptional target of c-Myc in SF188 and MRC5 cells through binding at an E-box in the promoter region [6]. Interfering with p32 expression in glioma cells reduced their sensitivity to glutamine starvation, suggesting an association between p32, and to an extent, mitochondrial respiration, and glutamine metabolism [6]. In fact, the loss of p32 in c-Myc-driven glutamine-addicted cells reverted the metabolic dependency from glutamine back to glucose [6]. Overall, c-Myc is responsible for switching the mitochondrial metabolism from glucose dependency to glutamine dependency through the TCA cycle and subsequent oxidative phosphorylation (Figure 3).

## 9. c-Myc’s Regulation of the Conversion of Glutamine into Non-Essential Amino Acids

Besides TCA cycle anaplerosis, glutamine can be transformed into various NEAAs endogenously, such as asparagine, aspartate, alanine, serine, glycine, and proline [18]. In fact, Feist et al. have shown, by labeling the nitrogen groups of glutamine, high labeled fractions of alanine, aspartate, glutamate, and proline in MYC-overexpressing P493-6 cells, suggesting that c-Myc promotes the synthesis of these amino acids from glutamine [65].

As discussed previously, c-Myc has been shown to induce the expression of aspartate transaminases GOT1 [27,61] and GOT2 [5,24,61], alanine transaminase GPT2 [24,61] as well as PSAT1 [63]. The importance of the transaminases for survival and growth in the context of MYC activation has also been characterized in various cell lines [22,24,61]. Accordingly, c-Myc drives the metabolism of glutamine toward the synthesis of aspartate, alanine, and phosphoserine, thus supporting the intracellular pool and availability of NEAAs. Furthermore, Sun et al. have studied Hep3B and P493-6 cells to further understand the regulation of c-Myc on the serine biosynthesis pathway (SSP). In addition to PSAT1, both the transcript and protein levels of the phosphoglycerate dehydrogenase (PHGDH), phosphoserine phosphatase (PSPH), and serine hydroxymethyltransferases (SHMT1/2), all fundamental in the SSP and glycine synthesis, were significantly upregulated by MYC overexpression [64]. In fact, c-Myc was shown to transcriptionally upregulate PHGDH, PSAT1, and PSPH by directly binding to their promoters near the transcription start site [64]. Moreover, this overexpression of SSP genes was crucial for the sustained survival of Hep3B and hepatic adenocarcinoma SK-HEP-1 cells [64]. Thus, c-Myc is an important regulator not only of PSAT1 expression, but of all the genes involved in the SSP and glycine synthesis.

Moreover, c-Myc has been shown to promote proline synthesis from glutamine. The proline synthesis pathway (PSP) begins with pyrroline-5-carboxylate synthase (P5CS), a step during which glutamate is converted into pyrroline-5-carboxylic acid, the direct precursor to proline [18,73]. The second step of the PSP requires pyrroline-5-carboxylate reductase 1 (PYCR1) and yields proline as a final product [18,73]. In P493-6 cells, Liu et al. showed that there was an MYC-specific increase in the glutamine carbon and nitrogen incorporation into proline, suggesting increased PSP in the context of MYC activation [71]. Additionally, c-Myc expression positively correlated with the levels of proline anabolic enzymes P5CS and PYCR1, but negatively with those of the proline oxidase (POX) enzyme, also termed proline dehydrogenase [71]. c-Myc has also been positively correlated with PYCR1 expression in luminal B breast cancer, but not in the luminal A subtype, suggesting that the regulation of c-Myc on PYCR1 transcription is dependent on specific tumor types [74]. Contrary to P5CS and PYCR1, POX is the rate-limiting enzyme involved in proline catabolism [73]. POX is a known tumor suppressor as it induces apoptosis and cell cycle arrest through the generation of reactive oxygen species [71,73]. Liu et al. showed that rather than directly binding at the promoter to regulate transcription, c-Myc interacts with POX through microRNA miR-23b* [71]. In fact, c-Myc upregulated the expression of Argonaute 2, involved in miR-23b* stability, and enhanced its binding activity to the 3′UTR of POX mRNA, leading to the degradation of the latter [71]. Finally, MYC amplification yielded cells dependent on proline anabolism and the concomitant suppression of proline catabolism for the optimal proliferation and survival of P493-6 and PC3 cells [71]. As such, c-Myc is a relevant and important regulator in directing glutamine toward the synthesis of NEAAs.

## 10. c-Myc’s Regulation of Glutathione Synthesis and Redox Balance

MYC-driven metabolic reprogramming has also been shown to have an impact on redox balance through glutathione (GSH). GSH is a tripeptide composed of glutamate, cysteine, and glycine, and is responsible for neutralizing reactive oxygen species (ROS) to attenuate cellular oxidative stress [51,75]. Through scavenging ROS, GSH is oxidized into GSSG dimers, which can be reduced back to GSH using high-energy NADPH [75]. Incidentally, the downstream metabolism of glutamine can provide all three amino acids that make up GSH. First of all, glutamate is the direct product of the first step of glutaminolysis [51]. Glycine is a product downstream of the SSP, as explained earlier, where glutamate is converted to L-serine, which is converted to glycine by the SHMT1/2 enzymes [18,60]. As for cysteine, glutamate is required as an exchanger to import cystine, the oxidized dimer of cysteine, at the plasma membrane [51,75]. In fact, the efflux of glutamate is coupled with the influx of cystine by the xCT (SLC7A11 gene) transporter, the latter being reduced back to cysteine in the cell under favorable redox equilibria [75].

Not unexpectedly, c-Myc has been shown to promote GSH synthesis. In Hep3B cells as well as human cholangiocarcinoma HIBEpic, HuCCT1, and RBE cell lines, c-Myc supported GSH production and recycling, such that the loss of c-Myc expression in these cells led to a decrease in GSH levels and GSH/GSSG ratios [64]. Furthermore, Xu et al. observed that inhibiting c-Myc by targeting its upstream positive regulator SIRT2, a histone deacetylase, led to increased ROS production [76]. Both studies proposed that this increase in GSH production was at least partially explained by the role of c-Myc in upregulating glycine synthesis SHMT1/2 enzymes as well as its capacity to stimulate SSP by increasing the transcription of all the SSP genes, as previously discussed [64,76]. With regard to cysteine, Tameire et al. have shown that c-Myc transcriptionally targets the SLC7A11 gene at its transcription start site in DLD-1 cells and promotes ATF4 to do the same [48].

On the other hand, Anderton et al. have found an opposite correlation between GSH and c-Myc in liver tumors [54]. In fact, GSH was among the most depleted metabolites observed under MYC overexpression, which, in turn, increased the sensitivity of cells to exogenous oxidative stress [54]. Incidentally, glutamate cysteine ligase (GCL), the rate-limiting enzyme of GSH synthesis, was markedly decreased in MYC-driven murine tumor samples, which contributed to decreased glutamine incorporation into GSH [54,75]. c-Myc was further described as a transcription factor of the oncogenic miR-17-92 cluster containing miR-18a, a known GCL repressor that targets the 3′ UTR [54,77]. Furthermore, c-Myc has been shown to activate the nuclear factor erythroid 2-related factor 2 (NRF2), a key player in redox balance and antioxidant mechanisms, downstream of KRAS activity [66,78].

Altogether, these findings suggest that the influence of c-Myc on GSH metabolism and oxidative stress in neoplastic cells is, at least under one perspective, dependent on the tissue of origin: c-Myc has been shown to have opposing effects on GSH levels in cholangiocarcinoma cells and hepatocarcinoma cells. Although c-Myc does support SSP and glycine synthesis, it suppresses the expression of GCL and also GSH synthesis in murine liver tumors. This interesting and seemingly paradoxical interaction between MYC and GSH needs to be studied further.

## 11. c-Myc’s Regulation of the Hexosamine Biosynthesis Pathway and Protein Glycosylation

Another of the many intracellular roles of glutamine is its incorporation in the hexosamine biosynthesis pathway (HBP). Hexosamines are a family of molecules composed of simple sugars and nitrogen groups [18]. Specifically, glutamine amide is used in the HBP, where it is conjugated with fructose-6-phosphate to form glucosamine-6-phosphate by glutamine:fructose-6-phosphate amidotransferase (GFPT1 gene) [18,79]. The final product of the HBP is uridine diphosphate N-acetylglucosamine (UDP-GlcNAc), which is used as a substrate for protein glycosylation [79,80]. During this process, proteins at serine and threonine residues are O-link GlcNAcylated by O-GlcNAc transferase (OGT) [79,80]. In the opposite direction, the removal of UDP-GlcNAc from proteins is catalyzed by O-GlcNACase (OGA) [79,80]. Proper O-GlcNAcylation is essential for protein homeostasis, such as proper folding and trafficking, which thus suppresses the endoplasmic reticulum stress response [18,80,81,82]. Glycosylation is implicated in protein expression and has been associated with cellular reprogramming such as epithelial-to-mesenchymal transition processes [79].

In isolated murine T lymphocytes, c-Myc has been shown to associate with increased levels of GFPT1 mRNA and protein [49]. Such a phenomenon suggests that c-Myc promotes HBP and the integration of glutamine amide toward the synthesis of UDP-GlcNAc. Furthermore, Morrish et al. have shown that global O-GlcNAcylation levels are increased exclusively in Rat1A fibroblasts with MYC activation [83]. Similar findings have supported a positive and specific correlation between c-Myc and OGT protein expression in multiple breast cancer cell lines, MDA-MB-231, SKBR-3, MCF-7, and SUM-159 [84]. In fact, Sodi et al. further described that the regulation of OGT by c-Myc required the expression of the c-Myc transcriptional target heat-shock protein 90 (HSP90), an inhibitor of protein ubiquitination [84]. In PDAC, c-Myc mediated the upregulation of the HBP genes downstream of KRAS [13]. Lastly, the HBP and O-GlcNAcylation were found to be critical for the growth, proliferation, and survival of MYC-overexpressing cells, such that the pharmacological inhibition of GFAT, the entry point of glutamine in the HBP, led to the growth arrest of MYC-positive, but not of MYC-negative, Rat1A fibroblasts [83].

These findings suggest an important role for the HBP and O-GlcNAcylation in the setting of metabolic reprogramming under c-Myc. In fact, the latter supports the flux of glutamine nitrogen toward the synthesis of UDP-GlcNAc and further supports OGT activity downstream of glucosamine synthesis. Interestingly, proper protein glycosylation through sustaining the HBP controlled by c-Myc is, in turn, essential for the proliferative phenotype of MYC-driven cells.

## 12. c-Myc’s Regulation of Glutamine Amide Flux toward De Novo Nucleotide Synthesis

The metabolism of glutamine is of prime importance for the de novo synthesis of nucleotides. Nucleotides are composed of pentose sugars, nitrogenous bases, and triphosphate groups [85]. Thus, nucleotide biosynthesis requires the following three ingredients: carbon, nitrogen, and energy, all of which can be derived from glutamine [85]. Importantly, glutamine amide groups are used in the first steps of inosine monophosphate (IMP) and uridine monophosphate (UMP) syntheses, the respective precursors of purines and pyrimidines [86]. Through the amidophosphoribosyltransferase (PPAT) and phosphoribosylformylglycinamidine synthase (PFAS) enzymes, two amide groups from glutamine are used in the synthesis of IMP [86]. As for UMP, the trifunctional CAD protein (carbamoyl-phosphate synthetase 2, aspartate transcarbamylase, and dihydroorotase) utilizes one amide group from glutamine [86].

The previous sections have discussed the versatile role of c-Myc in regulating cellular metabolism. Hence, correlations between c-Myc and nucleotide biosynthesis are not surprising. Metabolomics analyses by Sun et al. have shown that the synthesis of both AMP and UMP is strictly dependent on the expression of c-Myc in Hep3B cells [64]. Likewise, Bott et al. observed that the c-Myc-dependent upregulation of GS expression, as previously described, increased glutamine flux toward nucleotide biosynthesis [77]. In PDAC cells, mutant KRAS induced the c-Myc activity, which subsequently enhanced nucleotide synthesis through the pentose phosphate pathway [87].

The enhanced transcription of CAD by c-Myc was first demonstrated by Miltenberger et al. over two decades ago [88]. More recently, the mRNA levels of PPAT, PFAS, and CAD have been shown to be dependent on c-Myc expression in murine MYC-induced renal cell carcinoma by Shroff et al. [27] as well as in rat embryonic fibroblasts by Liu et al. [89]. The same has been observed in P493-6 cells for PPAT and PFAS [89], in human melanoma SK-MEL-19 cells for PPAT [90], as well as in activated T lymphocytes for both PPAT and CAD [49]. In vivo, murine hepatocarcinogenesis induced by MYC activation has been shown to express upregulated PPAT mRNA [89]. Further characterization of this pathway by Liu et al. has identified PPAT, PFAS, and CAD genes as molecular targets of c-Myc, each having E-boxes, namely PPAT and CAD in their respective promoters, as well as in the first introns of PFAS and CAD [89]. In fact, PPAT and PFAS were among 11 genes within the purine and pyrimidine synthesis pathways found to be directly bound by c-Myc [89]. Thus, c-Myc promotes glutamine flux toward nucleotide biosynthesis by upregulating the expression of glutamine-utilizing biosynthetic genes PPAT, PFAS, and CAD in the IMP and UMP synthesis pathways.

## 13. Interactions between Intracellular Pathways: Glutamine, mTORC1, and c-Myc

An interesting characteristic of glutamine is its ability to stimulate cell signaling pathways, which broadens its role in cell physiology and pathophysiology. A major pathway regulated by glutamine is the mechanistic Target of Rapamycin Complex 1 (mTORC1) [91]. mTORC1, as its name suggests, is a multiprotein complex activated by a variety of stimuli, such as intracellular amino acids [91,92,93]. Glutamine and its downstream metabolite αKG have both been shown to stimulate mTORC1 activity, similarly to leucine that does so through leucyl tRNA synthetase [92]. mTORC1 is a major regulator of protein synthesis by phosphorylating the ribosomal subunit S6 kinase 1 (S6K1) and the eukaryotic translation initiation factor 4E (eIF4E) binding protein 1 (4E-BP1), leading to its dissociation from eIF4E [91,93,94,95]. Additionally, mTORC1 inhibits autophagy by suppressing the ULK complex in the initial steps of autophagosome formation [95]. These diverse cellular functions make mTORC1 a compelling actor in the regulation of cell survival, growth, and proliferation.

Interestingly, the c-Myc and mTORC1 pathways have been shown to converge. Csibi et al. have found that c-Myc levels are regulated by S6K1 and that the pharmacological inhibition of mTORC1 suppresses c-Myc expression and its subsequent transcriptional activity [96]. In murine embryonic fibroblasts, this decrease in c-Myc led to a concomitant increase in intracellular glutamine levels, glutaminolysis being suppressed as a result of c-Myc downregulation [96]. Furthermore, the interaction between eIF4B, downstream of mTORC1, and c-Myc translation occurs at its 5′ UTR, where unwinding is critical for translation [96]. Sodi et al. confirmed that c-Myc is an indirect target of mTORC1 in several breast cancer cell lines [84].

On the other hand, c-Myc has been shown to promote mTORC1 activity [41,49]. The deletion of c-Myc in T lymphocytes has been shown to impair mTORC1 kinase activity [49]. Likewise, in liver tumors, the phosphorylated targets of mTORC1 were increased in a c-Myc-dependent manner [41]. Considering the role of intracellular amino acid pools in mTORC1 activation, Liu et al. described that c-Myc transcriptionally targets the high-affinity glutamine transporters ASCT2 and Y^+^LAT2, leading to increased intracellular glutamine levels, which, in turn, stimulate mTORC1 [41]. This upregulation of mTORC1 activity was found to be critical for c-Myc-driven murine hepatocarcinogenesis [41]. Together, these findings suggest an interesting interplay and inter-regulation between c-Myc and mTORC1, two pathways strongly linked with glutamine metabolism, cell growth, and survival.

## 14. Conclusions

Overall, through over a decade of research, glutamine has become a focal point in characterizing the cellular metabolism driven by c-Myc. In fact, MYC-overexpressing proliferative cells, whether they be of neoplastic or non-neoplastic origin, exhibit a distinct metabolic phenotype in which glutamine is used as a major substrate for diverse biosynthetic and bioenergetic pathways. Namely, as a transcription factor, c-Myc drives the expression of key genes involved in glutamine metabolism, which is essential for the survival and proliferation of these cells. Interestingly, however, c-Myc controls gene expression through pathways other than direct transcriptional targeting. Many findings point toward the ability of c-Myc to regulate miRNAs that, in turn, control the stability and levels of a wide variety of gene transcripts. Thus, c-Myc acts as a major regulator of the intracellular role and metabolism of glutamine, from its synthesis to its degradation, but also in other non-metabolic functions such as mTORC1 signaling. Glutamine uptake and consumption have also been shown to be driven by c-Myc in multiple studies. c-Myc, by its regulation on glutamine metabolism, induces substantial glutamine-derived αKG synthesis that becomes available as a source of mitochondrial energy in proliferative cells. Likewise, c-Myc supports glutamine flux toward NEAA, hexosamine, GSH, and nucleotide biosynthesis pathways. Moreover, several findings support an intimate relationship between KRAS and MYC, which seem to cooperate and maybe even act interdependently in cancer cell metabolic reprogramming. Among the most interesting phenomena observed secondary to c-Myc overexpression is glutamine addiction, in which cells rely on extracellular glutamine to ensure their rapid division, growth, and survival. As c-Myc is overexpressed in a wide variety of cancer types, among which are the most prevalent and aggressive of all malignancies, studying c-Myc has revealed new characteristics and novel vulnerabilities of cancer cells from a metabolic point of view as well as the relation between metabolic reprogramming and oncogenic drivers. As such, several key players of glutamine metabolism are requisites for the growth of MYC-driven cancer cells: GLS1, transaminases, and GFAT being particularly compelling targets in the context of MYC oncogene activation. Together, this innovative field has the potential to identify key aspects of tumor formation and progression that could lead to the development of novel, more specific cancer therapies.
